# Root exudation under maize/soybean intercropping system mediates the arbuscular mycorrhizal fungi diversity and improves the plant growth

**DOI:** 10.3389/fpls.2024.1375194

**Published:** 2024-06-14

**Authors:** Shu Zhang, Shumin Li, Lingbo Meng, Xiaodan Liu, Yuhang Zhang, Shuchang Zhao, Haobing Zhao

**Affiliations:** ^1^ Resource and Environmental College, Northeast Agricultural University, Heilongjiang, China; ^2^ School of Geography and Tourism, Harbin University, Harbin, Heilongjiang, China

**Keywords:** soybean/maize intercropping, interspecific interactions, root exudates, AMF, soil nutrients

## Abstract

**Introduction:**

Maize/soybean intercropping is a common cropping practice in Chinese agriculture, known to boost crop yield and enhance soil fertility. However, the role of below-ground interactions, particularly root exudates, in maintaining intercropping advantages in soybean/maize intercropping systems remains unclear.

**Methods:**

This study aimed to investigate the differences in root exudates between intercropping and monocropping systems through two pot experiments using metabolomics methods. Multiple omics analyses were conducted to explore correlations between differential metabolites and the community of Arbuscular Mycorrhizal Fungi (AMF), shedding light on the mechanisms underlying the dominance of intercropping from the perspective of root exudates-soil microorganism interactions.

**Results and discussion:**

The study revealed that intercropping significantly increased the types and contents of root exudates, lowered soil pH, increased the availability of nutrients like available nitrogen (AN) and available phosphorus (AP), and enhanced AMF colonization, resulting in improving the community composition of AMF. Besides, root exudates in intercropping systems differed significantly from those in monocropping, with 41 and 39 differential metabolites identified in the root exudates of soybean/maize, predominantly amino acids and organic acids. The total amount of amino acids in the root exudates of soybean intercropping was 3.61 times higher than in monocropping. Additionally, the addition of root exudates significantly improved the growth of soybean/maize and AMF colonization, with the mycorrhizal colonization rate in intercropping increased by 105.99% and 111.18% compared to monocropping, respectively. The identified metabolic pathways associated with root exudates were closely linked to plant growth, soil fertility improvement, and the formation of AMF. Correlation analysis revealed a significant relationship (P < 0.05) between certain metabolites such as tartaric acid, oxalic acid, malic acid, aspartic acid, alanine, and the AMF community. Notably, the photosynthetic carbon fixation pathway involving aspartic acid showed a strong association with the function of Glomus_f_Glomerace, the dominant genus of AMF. A combined analysis of metabolomics and high throughput sequencing revealed that the root exudates of soybean/maize intercropping have direct or indirect connections with AMF and soil nutrients.

**Conclusion:**

This suggests that the increased root exudates of the soybean/maize intercropping system mediate an improvement in AMF community composition, thereby influencing soil fertility and maintaining the advantage of intercropping.

## 1 Introduction

Intercropping, a traditional agricultural technique, has gained global traction for its efficient resource utilization, encompassing sunlight, water, and nutrients (Brooker et al., 2015). This practice is pivotal in sustaining biodiversity and stability within farmland ecosystems while achieving high and consistent yields in agroecosystems (Hauggaard-Nielsen et al., 2008; Zhang et al., 2012). Although significant studies have shed light on the advantages concerning aboveground productivity and resource sharing in intercropping settings, below-ground interspecific interactions, present a more intricate picture that warrants further investigation. In recent years, greater emphasis has been placed on exploring the microecology of the soil, including soil nutrients, root exudates, and soil microorganisms (Li et al., 2014). Understanding plant-soil-microbe interactions is crucial in comprehending intercropping systems (Duchene et al., 2017). However, the specific role of these interactions in intercropping remains poorly understood.

The rhizosphere, recognized for its crucial role in nutrient cycling and supporting diverse microbial populations (Kuzyakov and Blagodatskaya, 2015), is the key zone of interaction between plants and the soil. It facilitates the transfer of materials between crops and their soil environment (Kuzyakov and Razavi, 2019). In this dynamic environment, root exudates serve as essential channels for the exchange of substances and signals between plants and soil (Coskun et al., 2017). Notably, plants allocate approximately 10% of their photosynthetically fixed carbon to generate root exudates, enriching the rhizosphere (Mohanram and Kumar, 2019). These secretions are instrumental in enhancing soil texture and chemistry while also attracting and nourishing specific advantageous microbial colonies. This enhances the soil conditions, promoting healthier plant growth (Pérez-Jaramillo et al., 2016). Soil microbes have the capability to directly utilize root exudates as carbon sources (Swenson et al., 2015), and this communication between plants and microbes are vital in maintaining the vitality of the rhizosphere microecosystem. Root exudates facilitate below-ground interactions among plant species and significantly contribute to the link between biodiversity and ecosystem function (Zhang et al., 2017).

Root exudates, are pivotal in augmenting the nutrient dynamics within soil, thereby optimizing nutrient acquisition and assimilation by vegetation (Nardi et al., 2002). The interaction between root exudates and plant species manifests in several ways: they can activate insoluble soil phosphorus (P) and trace elements, and can also form chelates with various metallic elements, such as calcium (Ca), thereby facilitating nutrient absorption and utilization by crops such as alfalfa, legume, etc (Dakora and Phillips, 2002; Shen et al., 2002; Landi et al., 2006). Fe-deficient cereals release iminocarboxylic acids to dissolve ferric compounds for uptake by cereal roots (Dakora and Phillips, 2002). Beyond their nutritional role, root exudates can also alleviate the adverse impacts of heavy metals on crops, modulate the rhizospheric microbial ecosystem, suppress plant diseases, and improve the physical and chemical conditions of the soil (Okubo et al., 2016; Karlen et al., 2019; Wang et al., 2020). Simultaneously, allelochemicals within these exudates can influence the root development and nutrient uptake of adjacent plants, thereby impacting their growth either positively or negatively (Liu et al., 2013; Hu et al., 2018).

Research demonstrates that root exudates selectively shape the microbial community of the rhizosphere, which varies across plant species, resulting in distinctive microbial profiles (Paterson et al., 2007). In turn, changes in this microbial community significantly affect root exudate production, soil material cycling, energy flow, and information transfer, ultimately impacting plant growth and development (Eisenhauer et al., 2012). Furthermore, a significant relationship exists between the variety and quantity of soil metabolites and the stimulation of microbial genetic activity (Chaparro et al., 2013).

AMF are key soil microbes, integral to plant-soil interactions in intercropping systems. The symbiosis between AMF and plants significantly extends the nutrient absorption capacity through a widespread mycelial network. This network enhances plant nutrient uptake efficiency (Ferrol et al., 2019). Additionally, mycorrhizal symbiosis assists in immobilizing approximately 5 billion tons of photosynthetic products annually into the soil, thereby stabilizing the carbon balance of the ecosystem (Bago et al., 2000). Moreover, mycorrhizal relationships are instrumental in enhancing the growth and productivity of host plants. This leads to higher agricultural yields and lessens the dependency on chemical fertilizers, thereby supporting both food production and ecological health (Raklami et al., 2019). AMF are also effective at reducing nutrient losses due to leaching or denitrification by optimizing nutrient uptake (Querejeta, 2017), which is vital for enhancing soil quality. However, limited research has been conducted on the interrelationship and regulation between AMF, root exudates, and their underlying mechanisms within intercropping systems. Given the crucial roles of AMF and root exudates in the soil ecosystems, our research aims to delve into the alterations in exudates and the interplay between root exudates and AMF in intercropping systems.

This investigation utilized pot experiments to assess the impacts of interspecific interactions on root exudates and AMF communities in a soybean/maize intercropping system. Metabolomics and high-throughput sequencing were employed for a comprehensive analysis. This study tried (1) to delineate the metabolic variances in root exudates between of soybean and maize in both intercropped and monocropped systems, (2) to assess the impact of root exudates on plant growth and the soil’s microbial environment, and (3) to examine the relationship between root exudates and AMF communities. By uncovering the underlying mechanisms behind the advantages of intercropping from the perspectives of microorganisms and metabolites, this research seeks to bolster the theoretical underpinnings for the ecologically sustainable development of intercropping practices in agriculture.

## 2 Materials and methods

### 2.1 Experiment design

#### 2.1.1 Experiment 1: impact of interspecific interactions on root exudates, AMF and plant growth

This study employed a one-way completely randomized experiment consisting of three treatments: soybean monocropping, maize monocropping, and soybean/maize intercropping. Each treatment had six replications that were randomly assigned. The pot experiment was implemented at the Horticultural Experiment Station of Northeast Agricultural University (45°03’N, 126°43’E), Harbin, China, utilizing soybean (Glycine max L.) seeds of the Dongnong-252 variety and maize (Zea mays L.) seeds of the Xianyu-335 variety. The experiment commenced on August 15, 2020. The pot (Diameter 23 cm, depth 8 cm) was divided into two parts from the middle, one for maize and the other for soybean, applying two distinct partition methodologies: (i) a solid root barrier to simulate sole cropping by obstructing root interactions, and (ii) a 30-μm nylon mesh allowing for mass flow and diffusion between species, mimicking intercropping scenarios. (**Supplementary Figure S1**). The root barriers experiment could ensure that two plant species occupied the same soil space whether monocropping or intercropping (plastic sheet barrier versus nylon mesh barrier) and were simultaneous planted and harvested, and the aboveground growth conditions are the same, so as to avoid the difference of root exudates caused by the influence of factors such as light on plant growth.

Soil was procured from an undisturbed mollisoil (0-20 cm layer) at the Acheng Experimental Site (44°04’N, 125°42’E) in Harbin City, China. The soil contained 17.2 g total organic carbon (TOC), 1.47 g total nitrogen (TN), 125 mg available nitrogen (AN), 67.8 mg available phosphorus (AP) and 123 mg available potassium (AK) per kg soil, and pH is 6.10. The soil was prepared by air- drying and sieving through a 2-mm mesh sieve. Each compartment of the pot was then loaded with 1.5 kg of this prepped soil. Basal fertilizers were mixed in soil and thoroughly mixed before sowing. The soil for maize was supplemented with 120 mg/kg N by applying 0.39 g urea (N 46%) in each compartment, while the soybean soil received 60 mg/kg N by applying 0.20 g urea (N 46%) in each compartment. Additionally, the soil was fertilized with 50 mg/kg P_2_O_5_ by applying 0.12 g Ca(H_2_PO_4_)_2_ (P_2_O_5_ 61%) in each compartment and 100 mg/kg K_2_O by applying 0.28g K_2_SO_4_ (K_2_O 54%) in each compartment. Basal nutrients in solution were added to soil at the following rates (mg/kg soil): MgSO_4_(317), FeSO_4_·7H_2_O (31), MnSO_4_·H_2_O (20), CuSO_4_·5H_2_O (25) and ZnSO_4_·7H_2_O (28).

Seeds of soybean and maize were sterilized with 15% hydrogen peroxide(H_2_O_2_) for 20 minutes and rinsed with sterile water thrice. Subsequently, the sterilized corn seeds were soaked in sterile water for 24 hours to promote germination. After 24 hours of soaking, the corn seeds were placed on rectangular filter paper with approximately 1-2 cm spacing between the seeds, and arranged with the navel facing downward. The seeds were then pregerminated in the dark in a dish at a temperature of 18°C for 8 hours per day, taking approximately 1-2 days for the corns to grow by about 1 cm, and then transplanted them to the compartment of pot. Initially, four soybean seeds and two maize seeds were sown in separate compartments; thinning was conducted to maintain two soybeans and one maize plant per compartment 10 days post-sowing. The plants received consistent irrigation and were cultivated in a greenhouse regulated at 20°C to ensure optimal growing conditions.

After a growth period of 60 days, the plants were harvested on October 14, 2020. Samples from both the plant’s aboveground tissues and the soil surrounding the roots were gathered to evaluate aboveground and belowground biomass, root morphological characteristics (such as length, surface area, and volume), soil physicochemical traits, and variations in mycorrhizal colonization and root exudation between the soybean and maize under different treatment conditions.

#### 2.1.2 Experiment 2: root exudate addition experiment—effects of root exudates on AMF and plant growth

To further verify the impact of root exudates in intercropping systems on crops growth and AMF, the experiment 2 was designed with exogenous addition of root exudates. The soil was the same as experiment 1, and the study comprised eight distinct treatments, each replicated four times. The treatments were arranged as follows: 1) Soybean grown alone with deionized water added (S_W); 2) Soybean grown alone with soybean intercropping root exudates added(S_RIS); 3) Soybean grown alone with maize monocropping root exudates added (S_RMM); 4) Soybean grown alone with maize intercropping root exudates added (S_RIM); 5) Maize grown alone with deionized water added (M_W); 6) Maize grown alone with maize intercropping root exudates added (M_RIM); 7) Maize grown alone with soybean monocropping root exudates added (M_RMS); and 8) Maize grown alone with soybean intercropping root exudates added (M_RIS) (**Supplementary Figure S2**).

The experiment commenced on August 15, 2021, with the sterilization and germination of soybean and maize seeds, following the method described in Experiment 1. The sprouted seeds were then planted in the pots (Diameter 12 cm, depth 6 cm), each containing 1.5 kg of the prepared soil, air-dried and sifted through a 2 mm mesh. Fertilizer and potting management were conducted according to the guidelines outlined in experiment1. After the crops grow for 10 days, keep 2 soybeans or 1 maize in each pot. The crops were cultivated in a greenhouse at 20°C. Additionally, separate pots were utilized for the cultivation of soybean and maize monocrops, as well as intercrops, while root exudates were collected from soybean monocrop, soybean intercrop, maize monocrops, and maize intercrops, respectively. From the 14th day after sowing (DAS), root exudates were collected (dynamic collection for 24 hours), and the collected root exudates (RIM, RMM, RIS, RMS) were added to the rhizosphere soil of soybean and maize respectively according to the experimental settings. Add 40 ml exudates to the respective treatment every 5 days, while an equal measure of deionized water served as a control in comparison treatments. On the 60th DAS, samples from plant biomass and rhizosphere soil were collected for examination. The assessments included analysis of both aboveground and belowground biomass, and evaluation of root architectural traits, such as root length, surface area, and volume. In addition, the study extended to measure soil physicochemical traits, and the levels of amino acids and organic acid, alongside appraising variations in AMF colonization under different treatment conditions.

### 2.2 Plant growth and soil physicochemical properties

After harvesting, the aboveground and underground biomass of soybean and maize, as well as their respective root morphological parameters were measured. The plant’s aerial sections were initially sterilized at 105°C for 30 minutes and then consistently dried at 65°C until achieving a stable weight for subsequent analysis. Roots were imaged using an Epson scanner set to professional mode, 16-bit grayscale, at a resolution of 600 dpi, with the images being recorded in.jpg format. The WinRhizo 2005 software was applied to analyze these images, extracting data on total root length, diameter, volume, and surface area for both crops. Post-scanning, root samples were dried at 65°C to a constant weight for further assessments. In parallel, soil samples were collected, and a subset was air-dried and sieved through a 2 mm mesh to evaluate soil characteristics. Soil analyses encompassed measurements of pH, TN, AN, AP, AK, and TOC. Soil pH levels were gauged using a PHS-3C Meter (Toledo) in a 2.5:1 water-to-soil ratio.TN levels were determined following the Kjeldahl distillation technique (Hou et al., 2007). AP levels were gauged using the sodium bicarbonate (NaHCO_3_) solution method at pH 8.5, following Olsen’s protocol (Olsen and Sommers, 1982). AN concentrations were obtained through the diffusion adsorption method (Lu, 2000). AK extraction was performed with NH_4_OAc (pH 7.0) and quantified using flame atomic absorption spectrometry (Schollenberger and Simon, 1945). TOC was determined utilizing the Multi N/C 2000 TOC analyzer (Germany), following the method of Xiao et al (Xiao et al., 2019). Finally, the mycorrhizal colonization rate was established utilizing the trypan blue staining technique (Giovannetti and Mosse, 1980).

### 2.3 AMF mycorrhizal infection rate and community

For determining the extent of AMF colonization in plant roots, we adapted the procedure described by Phillips and Hayman (1970). Fine roots (2 mm in diameter) were cut into segments of 1-2 cm length and subjected to clearing with 10% KOH at 90°C for 30 min, followed by triple rinsing with distilled water. The root segments were then acidified using 2% HCl and stained with a 0.05% trypan blue solution for 15 min at 90°C, followed by another series of washes with distilled water. Subsequently, the stained roots were immersed in a lacto-glycerol mixture (equal parts of lactic acid, glycerol, and water) and allowed to stand at ambient temperature. For microscopic examination, 15 root fragments were positioned on slides pre-marked for analysis (resulting in 60 fragments evaluated per treatment) (Brundrett and Kendrick 1990). Observations were conducted under 40× and 100× magnification. The degree of AMF infestation in root segments was categorized into 0, 10%, 20%…100% infestation levels, and the mycorrhizal infestation rate (%) of the samples was calculated by the weighting method of root infection rate.

The rhizosphere soil AMF community composition at genus level has been measured by high-throughput sequencing. Nested PCR was utilized to amplify fragments of the arbuscular mycorrhizal fungal 18S rRNA genes. The first PCR amplification was performed utilizing the universal primers AML1F (5’-ATCAACTTTCGATGGTAGGATAGA-3’) and AML2R (5’-GAACCCAAACACTTTGGTTTCC-3’). For the second PCR procedure, we used these amplicons from the first PCR as the template, and the AMF-specific primers AMV4-5NF (5’-AAGCTCGTAGTTGAATTTCG-3’) and AMDGR (5’-CCCAACTATCCCTATTAATCAT-3’) were used to amplify the partial AMF 18S rRNA gene fragment (Xu et al., 2017). The resultant products from the second PCR, following verification and purification, were sequenced employing the Illumina MiSeq platform from Majorbio Bioinformatics Technology Co., Ltd. (Shanghai, China). Subsequently, sequencing outputs were consolidated and subjected to quality checks utilizing FLASH software. Clustering of the sequences into operational taxonomic units (OTUs) was implemented at a similarity threshold of 97%. Taxonomic classification of all sequences into diverse groups was executed utilizing the RDP Classifier algorithm against the MaarjAM database with a confidence threshold of 70% (Öpik et al., 2010).

### 2.4 Soil metabolite analysis

#### 2.4.1 Collection and extraction of root exudates

After 60 days of plant growth, collect soil solution from the rhizosphere of crops and use a soil solution sampler (Rhizon soil moisture samplers™, OD 2.5mm, nylon wire, Pore size of the membrane 0.12, μm PVC-PE tubing 30 cm; Rhizosphere Research Products bv Wageningen, Netherlands) as an instrument. Before use, the samplers were washed using de-ionized water and dried at 25°C. Gently insert the sampler into the rhizosphere soil 5-8 cm away, connect the outer end of the sampler to a syringe, and then vacuum the inside of the syringe. Use pressure to suck the soil solution into the syringe, Disposable syringes of 50ml were used at selected sampling dates to collect 40 ml soil solution, collecting five times in total. To collect the soil solution, soil moisture was kept at 70% capacity. During the collection process, promptly store the collected solution in a -80°C refrigerator, and mix and store the solutions in each basin after they are fully collected. Once collected, the root secretions were concentrated to a volume of 50 mL. To analyze the amino acids, 35 mL of the filtrate was vacuum freeze-dried to obtain a dry powder, which was then dissolved in 0.5 mL of deionized water. The dissolved powder was transferred to a 1.5 mL centrifuge tube and stored in a -20°C refrigerator for further analysis. Furthermore, for the analysis of low molecular weight organic acids, 15 mL of the filtrate was vacuum freeze-dried to obtain a dry powder, which was then dissolved in 1 mL of deionized water. The solution was placed in an ice bath at -20°C for analysis.

#### 2.4.2 Nontargeted measurement of root exudates

The method for extracting soil metabolites was modified from Swenson and Northen’s technique (Swenson and Northen, 2019). Briefly, one gram of soil was transferred into a 5 ml EP tube, to which 1 ml of methanol (adjusted to a 3:1 volume/volume ratio with water) and 1 ml of ethyl acetate were added, along with 10 μl of adonitol (0.5 mg/ml) serving as an internal standard. This blend was homogenized utilizing a ball mill at a frequency of 45 Hz for 4 min, followed by an ultrasonic bath in chilled water for 5 min. The mixture was then centrifuged at 12,000 rpm and 4°C for 15 min, and the supernatants were collected into fresh EP tubes. This extraction step was repeated once more with another 1 ml of methanol and 1 ml of ethyl acetate. Post a second cycle of homogenization and centrifugation, the collected supernatants were merged and subsequently dehydrated using a vacuum concentrator at ambient temperature. To finalize the sample preparation, 30 μl of methoxyamine hydrochloride (dissolved in pyridine to make a20 mg/ml solution) was introduced to the desiccated extracts, followed by a 30-minute incubation at 80°C.

Non targeted analysis of metabolites was carried out using the LC-MS system. Chromatographic resolution occurred through a UPLC system by SCIEX (UK) (Seybold et al., 2020). For separation, an ACQUITY UPLC T3 column (100 mm × 2.1 mm, 1.8 μM, Waters, UK) was utilized under reversed-phase conditions. The column temperature was set at 35°C with a consistent flow of 0.4 mL/min. Gradient elution was conducted utilizing a mixture of solvent A (water enhanced with 0.1% formic acid) and solvent B (acetonitrile enhanced with 0.1% formic acid), based on the modified approach by Yu and colleagues (Yu et al., 2018). An injection volume of 4 μL was used for the analysis. ESI-MS^n^ evaluations were performed with a Thermo Q Exactive mass spectrometer, scanning both positive and negative ion modes. Settings for the mass spectrometer were as follows: mass range 50–1200 Da; spray temperature 400°C; ion source temperature 120°C; nebulizer gas flow 800 L/h; and capillary voltage 40 V. Identification of soil metabolites was completed by Majorbio BioPharm Technology (Shanghai, China), and the subsequent data handling was performed employing the Majorbio Cloud Platform (www.majorbio.com).

#### 2.4.3 Targeting measurement of specific root exudates

##### 2.4.3.1 Amino acid analysis

Amino acids in the analyzed root exudates were quantified using high-pressure liquid chromatography with pre-column derivatization (Li et al., 2013). The amino acids were initially converted into fluorescent compounds, followed by separation on a C_18_ column. Detection and quantification were achieved using fluorescence detection methods, supported by comparative analyses with standard amino acid mixtures. The chromatographic conditions included the use of a Waters 470 fluorescence detector and a Nova2pak C_18_ column (150 × 3.9 mm, 4 μm) at ambient temperature. The mobile phase comprised two solvents: solvent A (20 mmol/L sodium acetate solution) and solvent B (a blend of 20 mmol/L sodium acetate solution, methanol, and acetonitrile in a 1:2:2 ratio, v/v/v). Fluorescence detection settings included an excitation wavelength of 338 nm, and an emission wavelength of 262 nm. The application of a gradient elution method was utilized with a flow rate set at 1.0 mL min^-1^, and the injection volume was fixed at 10 μL. Amino acid identification was based on matching the mass-to-charge ratio (*m/z*), retention time, and ESI−MS/MS dissociation patterns to those of authentic standards.

##### 2.4.3.2 Organic acid analysis

The type and mass concentration of organic acids were analyzed utilizing high-performance liquid chromatography (HPLC). The HPLC method employed the LC-100 high-performance liquid chromatograph, which consisted of an LC-P100 high-pressure constant flow pump, an LC-UV100 ultraviolet detector, an autosampler, and a temperature-controlled box.

The chromatographic setup utilized for analysis incorporated a reversed-phase C_18_ column (150 mm × 4.6 mm, 5 μm), employing an 18 mmol/L KH_2_PO4 (pH 2.48), as the mobile phase. The operational temperature of the column was maintained at 30°C, and the mobile phase flow rate was established at 1.0 mL/min. For each run, a 10 μL sample volume was introduced, and analyte detection was conducted at 214 nm, with an overall run time of 15 min. The identification of LMWOA relied on comparing their retention times with those of standard organic acids. Quantification of the organic acids present in the samples was achieved through the external standard technique, with concentration calculations based on the comparative peak areas.

### 2.5 Data and statistical analysis

Principal component analysis (PCA) was implemented utilizing the SIMCA 14.1 software (Umetrics, Umeå, Sweden). For bubble diagram analysis, MetaboAnalyst was utilized (Chong et al., 2019). We analyzed the significance of differences in metabolites utilizing analysis of variance (ANOVA) and applied the least significant difference (LSD) tests for *post hoc* comparisons in SPSS V17.0, setting the significance threshold at p < 0.05. Orthogonal partial least squares discriminant analysis (OPLS-DA) was conducted with variable importance (VIP) values of N1. The relationships and visual representations of the differences between microbes and metabolites were explored utilizing R software v.3.2.1 and Cytoscape 3.4.0.

## 3 Results

### 3.1 Analysis of differential metabolites between monocropping and intercropping systems

**Figure 1:**
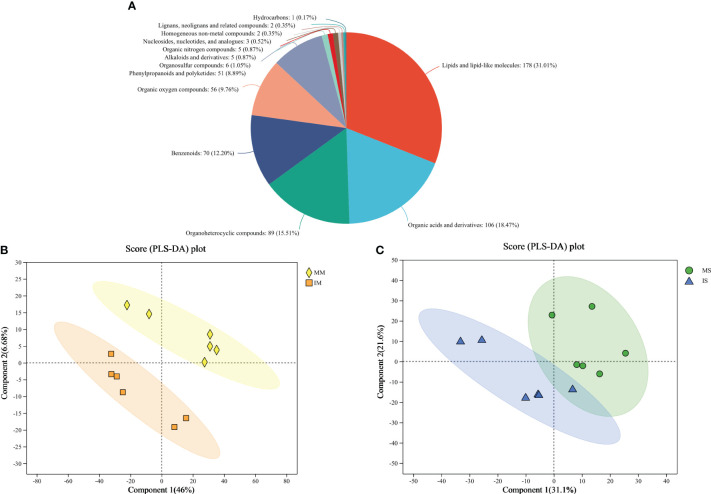
Metabolites types identified from monocropping and intercropping of soybean and maize root exudates **(A)**. Partial Least-Squares Discriminant Analysis of the root exudates in maize **(B)** and soybean **(C)** soils.

Untargeted metabolomic profiling of the root exudates revealed a diverse array of compounds. A total of 13,885 mass spectrum peaks were detected from the root exudates, with 749 metabolites identified. Among these metabolites, 574 were found in public databases such as HMDB and Lipidmaps. The secreted compounds were further categorized, including 178 lipid substances, 106 organic acids, 89 organic heterocyclic compounds, 70 benzene-type compounds, 56 organic oxygen compounds, 51 phenylpropane and polyketone compounds, as well as other compounds. The proportions of these various compound classes were 31.01%, 18.47%, 15.51%, 12.20%, 9.76%, 8.89%, and 4.18% respectively (**Figure 1A**).

The PLS-DA plot comparing MM *vs* IM and MS *vs* IS were presented in **Figures 1B, C**. As observed from the figure, a clear distinction can be made between MM and IM as well as MS and IS, suggesting significant variations in the content of different compounds in the root exudates of soybean and maize under monocropping and intercropping conditions (**Figures 1B, C**).

**Figure 2:**
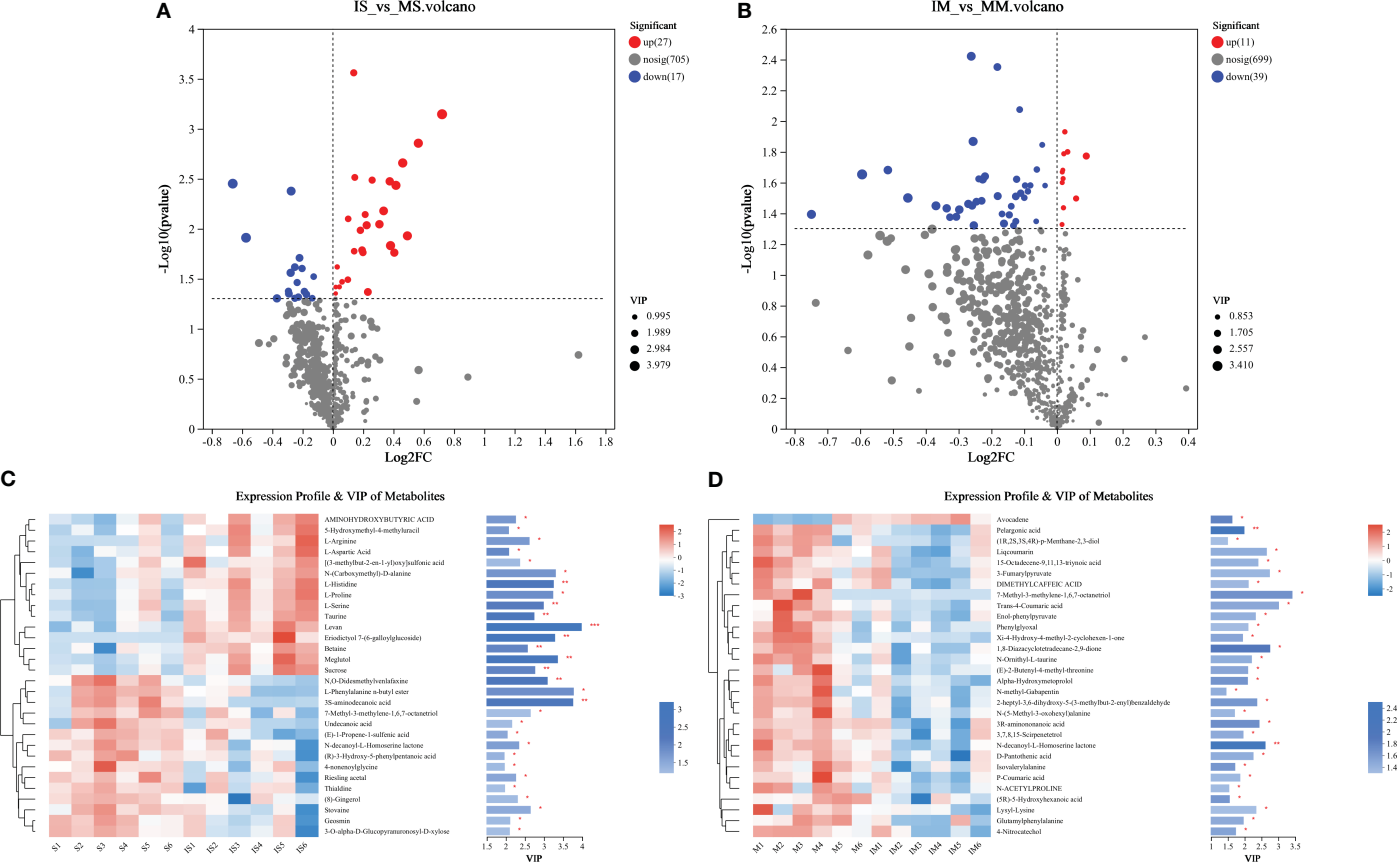
Volcano map **(A, B)** and Heatmap map (Top30) **(C, D)** of metabolites expressed differently between “IM *vs* MM” and “IS *vs* MS”.

Based on the ANOVA of known metabolites, significant differences (*P* < 0.5) were witnessed in the mean concentrations of 39 and 41 metabolites between IM and MM, as well as between IS and MS soil types (**Figure 2**). Examining the species composition of different metabolites, it was found that 38 root exudates exhibited significant up-regulated changes (**Figures 2A, B**). Further investigation into the root exudates with significant differences between intercropping and monocropping in maize identified eleven specific up-regulating substances, including Avocadiene, Phytal, 3,6,7-Trihydroxy-4’-methoxyflavone 7-rhamnoside, Acetyl tributyl citrate, Myristoleic acid, Shogaol, Gluten exorphin, 2-amino-14,16-dimethyloctadecan-3-ol, PE, and PG. Similarly, the analysis of common and unique root exudates in intercropping and monocropping of soybean revealed 27 specific up-regulating substances, primarily consisting of amino acids and their derivatives (9), sugars and their derivatives (3), small molecular acids (4), alcohols (2), amines (1), alkaloids and their derivatives (1), and others (7). These findings indicate that intercropping significantly alters root exudates.

**Figure 3:**
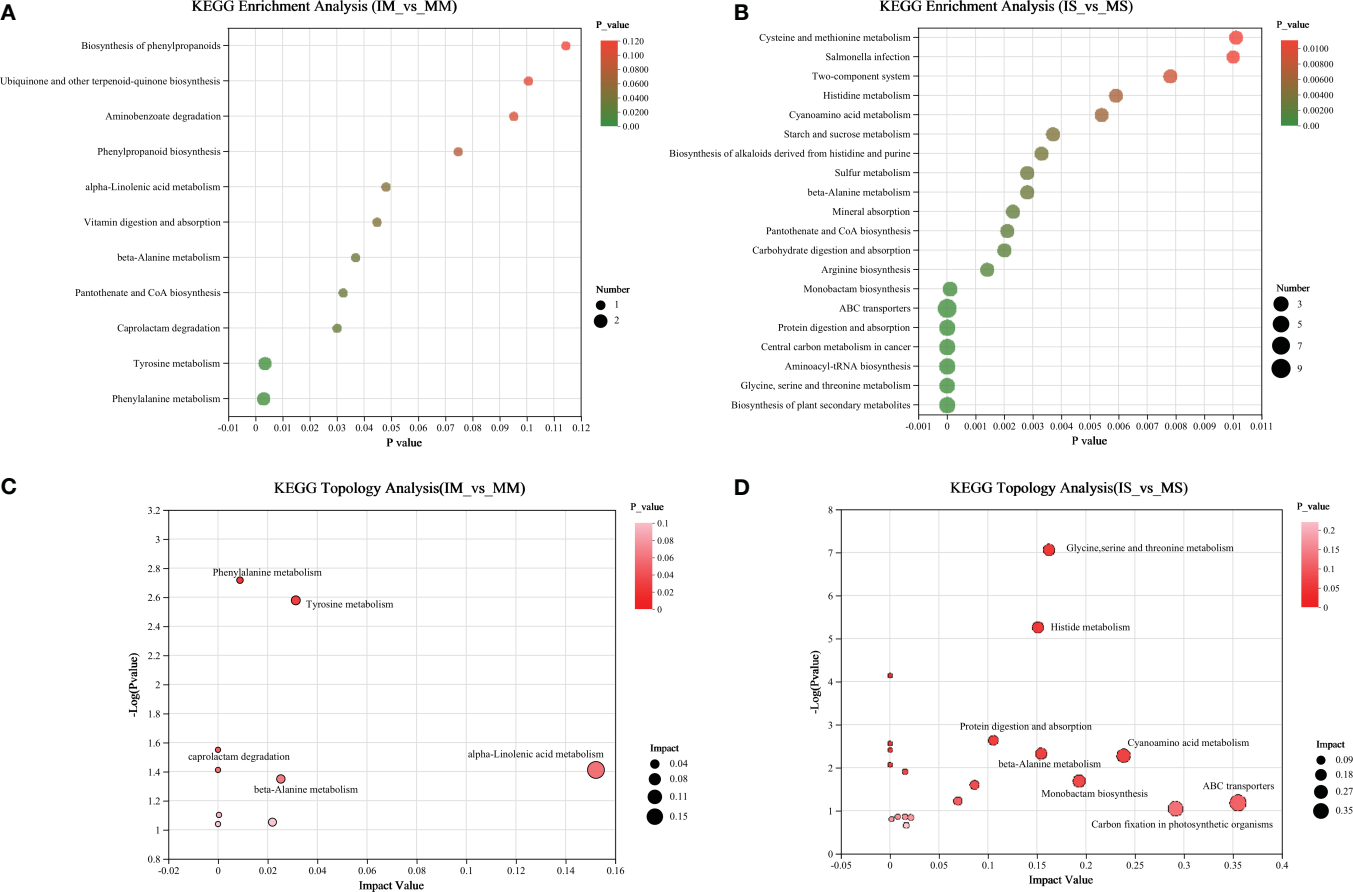
KEGG functional pathway involved in different metabolites of “IM&MM” **(A)** and “IS&MS” **(B)**. Topological diagram of important functional paths participated by differential metabolites in “IM&MM” **(C)** and “IS&MS” **(D)**.

To elucidate the primary metabolic pathways associated with differential metabolites, all identified metabolites in the root exudates of maize/soybean monocropping and intercropping were imported into the online analysis platform, Metaboanalyst (http://www.metaboanalyst.ca/). We identified significant pathways based on an impact value threshold above 0.1 and an enrichment pathway p-value below 0.05 (**Figure 3**). In the comparison between IM and MM, five distinct pathways were characterized: Phenylalanine metabolism, Tyrosine metabolism, beta-alanine metabolism, Caprolactam degradation, and alpha-linolenic acid metabolism (**Figures 3A–C**). Likewise, in the comparison between IS and MS, five distinct pathways were identified: Glycine, serine, and threonine metabolism, beta-alanine metabolism, Cyanoamino acid metabolism, Histidine metabolism, and Carbon fixation in photosynthetic organisms (**Figures 3B–D**).

### 3.2 The effect of intercropping on the content of amino acids and organic acids in root exudates

Quantitative targeted analysis of amino acids in the root exudates of monocropped and intercropped soybeans revealed notable findings. Glu and Arg were solely detected in the root exudates of soybean intercropping, whereas Asp, Thr, Ser, Gln, Gly, Ala, Val, Ile, Tyr, Phe, Lys, and His displayed significantly higher levels in intercropped soybeans compared to monocropped soybeans (*P* < 0.05). The total amount of amino acids in the root exudates of intercropped soybeans was 3.61 times greater than that in monocropped soybeans (**Table 1**). In this study, it was found that soybean/maize intercropping significantly improved the exudation of amino acids by soybean roots. Furthermore, aspartic, glutamic, alanine and other amino acids also participated in important metabolic pathways, such as alanine metabolism and glutamate metabolism, which promote plant growth.

**Table 1:** Content of amino acids and organic acids in root exudates under soybean/maize monocropped and intercropped system (unit: ug/L).

	IS	MS	IM	MM
Asp	47.70 ± 3.85a	14.10 ± 3.63b	17.99 ± 3.28a	15.73 ± 0.04a
Thr	46.87 ± 4.19a	14.52 ± 2.60b	14.82 ± 5.19a	13.88 ± 3.38a
Ser	214.17 ± 51.92a	49.99 ± 7.82b	54.61 ± 22.12a	54.16 ± 8.86a
Glu	24.60 ± 6.21	——	8.20 ± 1.44a	3.61 ± 0.01b
Gln	25.30 ± 1.60a	7.16 ± 1.30b	16.34 ± 4.19a	11.52 ± 3.65b
Gly	100.26 ± 28.80a	25.08 ± 6.50b	27.52 ± 3.19a	25.88 ± 2.41a
Ala	68.10 ± 2.36a	20.11 ± 6.62b	22.00 ± 5.47a	19.64 ± 2.43b
Val	34.91 ± 5.61a	17.40 ± 3.11b	21.27 ± 5.45a	17.50 ± 0.25b
Cys	1.53 ± 0.14a	1.74 ± 0.08a	——	0.43 ± 0.01
Met	5.15 ± 1.17a	3.49 ± 0.42a	——	——
Ile	18.74 ± 3.97a	7.28 ± 1.74b	8.99 ± 2.25a	7.37 ± 0.54a
Leu	22.98 ± 2.86a	9.73 ± 0.94b	7.53 ± 1.77a	9.79 ± 1.67a
Tyr	21.76 ± 1.74a	13.13 ± 4.06b	9.66 ± 3.08a	6.07 ± 4.60b
Phe	16.75 ± 3.68a	5.91 ± 1.57b	4.88 ± 0.71a	5.84 ± 1.18a
Lys	24.73 ± 5.82a	6.13 ± 0.90b	13.12 ± 3.53a	10.47 ± 1.70b
His	79.45 ± 13.19a	13.87 ± 2.17b	19.09 ± 5.73a	9.12 ± 0.92b
Arg	3.29 ± 1.90	——	12.48 ± 5.90	——
Total amino acid	756.27 ± 89.58a	209.64 ± 40.70b	258.47 ± 52.95a	211.00 ± 20.62b

Different lower-case letters in the same row indicate significant differences in different treatments (P < 0.05).

Targeted quantitative assessments of amino acids in maize root exudates from both monocropped and intercropped systems yielded significant insights. Specifically, Arg was uniquely identified in the exudates from intercropping, whereas Cys was exclusively observed in those from monocropping. Additionally, concentrations of Glu, Gln, Ala, Val, Tyr, Lys and His were found to be elevated in the exudates of intercropped maize compared to those of monocropped maize (*P* < 0.05). The aggregate level of amino acids in the exudates from intercropped maize exceeded that from monocropping by 1.22-fold. Importantly, glutamine, tyrosine, and alanine are essential components participating in key metabolic pathways such as β-alanine metabolism, caprolactam degradation, and tyrosine metabolism.

Notable variations were identified in the organic acid content of both crops under monocropping and intercropping conditions (**Table 2**). The total amount of organic acids in soybeans from intercropped settings markedly exceeded that of those grown in isolation, reaching a magnitude 1.80 times greater. Notably, oxalic acid (442.86%, *P* < 0.05), malic acid (185.71%, *P* < 0.05), and lactic acid (31.61%, *P* < 0.05) exhibited significantly higher levels in intercropping than corresponding monocropping, and citric acid was not detected in soybean root exudates. Conversely, maize displayed an augmented total organic acid concentration under monocropping rather than under intercropping, with notable increases in oxalic (172.22%, *P* < 0.05) and malic acids (200.00%, *P* < 0.05) when grown alone. However, intercropped maize showed higher quantities of tartaric, lactic (12.59%, *P* < 0.05), and citric acids (300.00%, *P* < 0.05) than when grown singly.

**Table 2:** Content of organic acids in root exudates under soybean/maize monocropped and intercropped system (unit: mg/L).

	IM	MM	IS	MS
tartaric acid	0.07 ± 0.00a	0.06 ± 0.00a	0.08 ± 0.00a	0.06 ± 0.00a
oxalate	0.18 ± 0.06c	0.31 ± 0.09b	1.14 ± 0.50a	0.21 ± 0.07c
malic acid	0.20 ± 0.10b	0.60 ± 0.22a	0.60 ± 0.27a	0.21 ± 0.11b
lactic acid	1.61 ± 0.30c	1.43 ± 0.21d	2.54 ± 0.21a	1.93 ± 0.68b
citric acid	0.04 ± 0.01a	0.01 ± 0.00b	——	——
Total organic acid	2.08 ± 0.48c	2.40 ± 0.07b	4.35 ± 0.57a	2.41 ± 0.84b

Different lower-case letters in the same row indicate significant differences in different treatments (P < 0.05).

### 3.3 Effects of root exudates on plant growth and AMF colonization in intercropping

**Figure 4:**
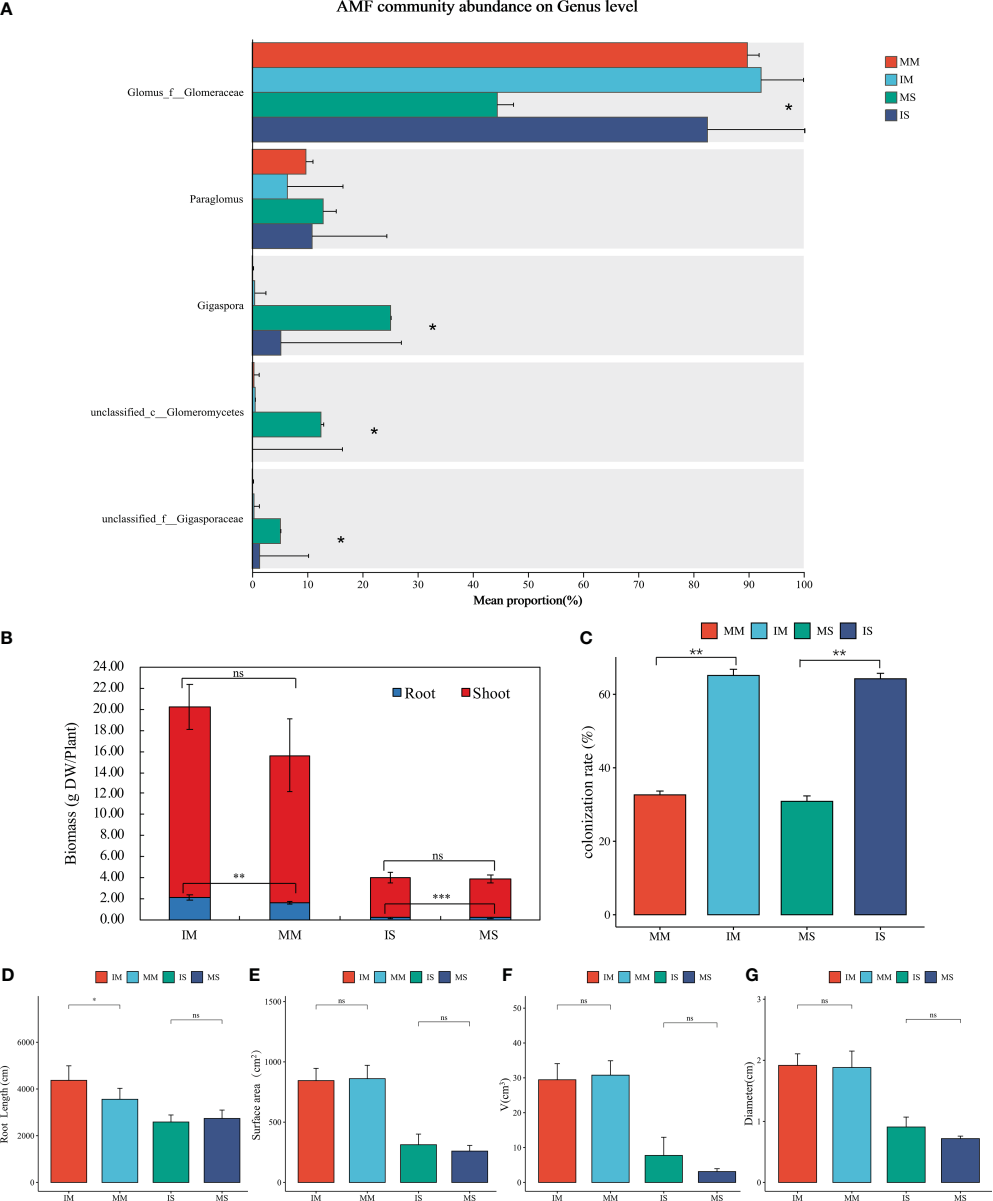
Effects of interspecific interaction on plant growth and AMF in intercropping system. Including: Relative abundances of main AMF genus in the rhizosphere soil of soybean/maize monocropping and intercropping **(A)**, The aboveground and underground biomass **(B)**, AMF colonization rate **(C)** and Root morphological parameters **(D–G)** of maize/soybean under different planting methods. *Represents the significant difference between different treatments at P < 0.05, ** represents the significant difference between different treatments at P < 0.01, *** represents the significant difference between different treatments at P < 0.001, ns represents no significant difference between different treatments.

Although not statistically significant, the biomass of soybean/maize plants separated by nylon net was higher than those separated by plastic. However, a significant difference was witnessed in the biomass of the roots, with the roots separated by nylon net displaying significantly higher biomass than those separated by plastic (*P* < 0.05). This underground interaction resulted in a remarkable elevation in the biomass of maize roots by 30.30% and soybean roots by 52.63% (**Figure 4B**). Compared to plastic separation, nylon net separation enhanced the exchange of root exudates and nutrients in the underground environment, facilitating biomass accumulation in both soybean and maize plants.

Throughout the development phases of soybean and maize, the incorporation of root exudates was observed to notably enhance both aboveground and belowground biomass, surpassing the results from the control group devoid of root exudate supplementation (**Supplementary Figures S3A, B**). The dry weight of soybean added deionized water and added root exudates of IS, MM and IM were 2.42 g, 3.10 g, 2.67 g and 3.68 g, respectively. Adding root exudates of IS, MM and IM increased the dry weight of soybean shoot by 35.22%, 23.99% and 47.29% respectively, and the dry weight of soybean root increased by 42.74%, 51.28% and 66.67%, respectively. It can be seen that the application of IS and IM root exudates has a more significant increase in aboveground biomass, and the application of IM root exudates has a more significant increase in root biomass. This effect underscores the significance of specific compounds within root exudates in fostering both vegetative and root growth. Moreover, the infusion of root exudates, particularly those derived from maize within an intercropping context, resulted in a marked increase in the formation of soybean nodules, underscoring the vital role these exudates play in augmenting soybean nodulation (**Supplementary Figure S3E**).

The root length of maize under nylon net separation was significantly higher than that of maize under plastic separation by 22.76% (*P* < 0.05, **Figures 4D–G**). When separated by plastic, crops underground growth mode is equivalent to monoculture, while under nylon mesh separation, Crops can interact through root exudates between each other. This implies that root exudates have a promoting effect on root growth under the same aboveground condition. Additionally, the adding of root exudates brings about the enhancement of root length and surface area in soybean and maize roots, as depicted in **Supplementary Figures S3F–M**. For example, the root length of soybean was increased by 61.60% with added root exudates of IM, and the root length of maize was increased by 34.11% with added root exudates of IS. This showed that root exudates play an important role in soybean/maize intercropping system and have a direct effect on plant root growth.

The results revealed a significantly higher AMF colonization rate in soybean/maize intercropping compared to their respective monocropping (*P* < 0.05). Specifically, the mycorrhizal colonization rate in intercropping increased by 105.99% and 111.18% when compared to monocropping conditions, as depicted in **Figure 4C**. Moreover, in this experiment, intercropping significantly increased the relative abundance of *Glomus_f_Glomeraceae*, the dominant genus of AMF (**Figure 4A**). *Glomus* plays an important roli in soil carbon fixation and nutrient cycling. So, it is suggested that intercropping not only enhances the colonization rate of arbuscular mycorrhizal fungi, but also improves the community of AMF. Moreover, the addition of root exudates from soybean or maize during the growth process significantly increased the AMF colonization rate compared to treatments without root exudates, as shown in **Supplementary Figures S3C, D**. In the growth of soybean, the mycorrhizal infection rate of the treatment group added root exudates of maize intercropping was the highest, which was 126.11% higher than that without root exudates. During the growth of maize, the mycorrhizal infection rate of the treatment added root exudates from soybean intercropping was the highest, which was 97.38% higher than that without root exudates. It is worth noting that adding root exudates from soybean intercropping significantly increased mycorrhizal infection in soybeans, while adding root exudates from maize intercropping did not have a significant effect on mycorrhizal infection in maize. This observation suggests that intercropped system mediated an increase in the type and quantity of root exudates, and certain substances in which may facilitate mycorrhizal colonization and community improvement.

### 3.4 Correlation between root exudates and AMF community

**Figure 5:**
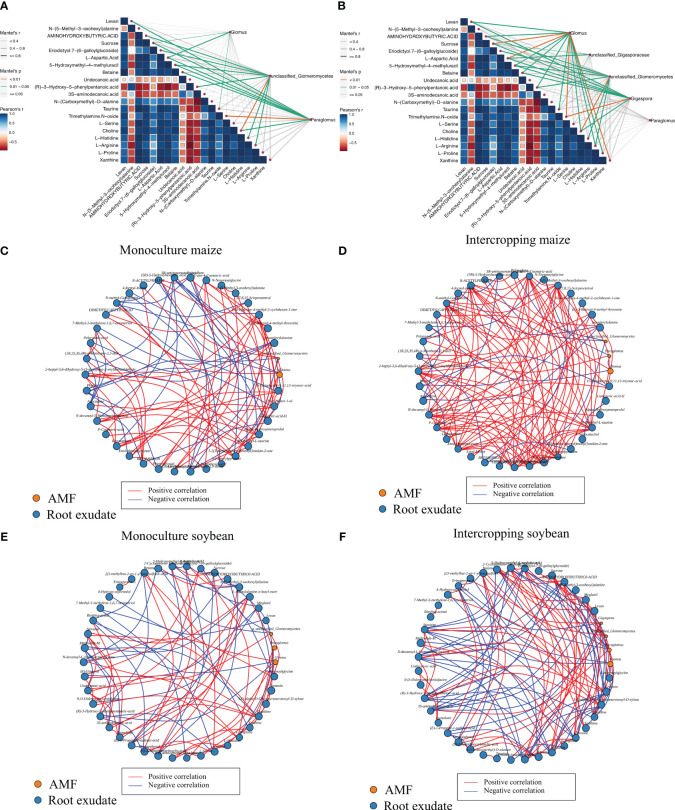
Correlation analysis between AMF and different metabolites of maize **(A)** and soybean **(B)** in monocropping and intercropping system. Analysis of the interaction network between differential metabolites and AMF (genus level) in maize monocropping **(C)** and intercropping **(D)**, and soybean monocropping **(E)** and intercropping **(F)**.

Utilizing the Mantel and Procrustes analyses, correlations were assessed between the top 20 distinct metabolites identified in the root exudates and the composition of the AMF community, as depicted in **Figures 5A, B**. Notable associations emerged between certain metabolites and the mycorrhizal fungal community within both monocropped and intercropped systems. Specifically, Within the differential metabolites between soybean monocropping and intercropping, several metabolites, including amino acids like L-Proline, L-Histidine, and L-Arginine, the saccharide Levan, certain organic acids such as Taurine and (R)-3-hydroxyl-5-phenylpropanoic acid, the biomolecule Choline, and the nucleic base analog 5-Hydroxymethyl-4-methyluracil were significantly correlated (*P* < 0.05) with *Glomus*, the predominant genus of the AMF community. Furthermore, compounds like Sucrose, L-Aspartic Acid, Trimethylamine N-oxide, and Xanthine were highly significant correlated (*P* < 0.01) with *Glomus* in soybean soil. Trimethylamine N-oxide also showed a positive association (*P* < 0.01) with *Gigaspora*. In contrast, within maize environments, both Sucrose and L-Aspartic Acid demonstrated notable correlations (*P* < 0.05) with *Glomus*, while Trimethylamine N-oxide was closely linked (*P* < 0.05) with the *Paraglomus* genus, and Choline was highly significant correlated (*P* < 0.01) with *unclassified_c_Glomeromycetes*.

The differential metabolites obtained from the intercropping and monocropping systems of soybean and maize were analyzed for their interaction networks with important genera of AMF. The results, illustrated in **Figures 5C–F**, demonstrated that the intercropping of soybean and maize led to a more intricate interplay between root exudates and AMF genera. In the interaction network specific to soybean monocropping (**Figure 5E**), there were 42 nodes, consisting of 39 differential metabolites and interactions with three AMF genera. This network comprised 83 pairs of interactions, with 47 pairs showing positive correlations and 36 pairs exhibiting negative correlations. The root exudates with the highest number of interactions were 3S-amino decanoic acid (amino acids) and L-Histidine (amino acids), while the genus with the most complex interactions was *Glomus*. Similarly, the soybean intercropping interaction network (**Figure 5F**) consisted of 43 nodes, 39 differential metabolites, and interactions involving four AMF genera. It encompassed 122 pairs of interactions, of which 63 pairs exhibited positive correlations, and 59 pairs displayed negative correlations. The root exudates that demonstrated the most complex interactions with the AMF community in this network included (R)-3-Hydroxy-5-phenylpentanoic (organic acids), Geosmin (alcohols), Histidine (amino acids), and Eriodictyol 7-(6-galloylglucoside) (sugar). Furthermore, the maize monocropping interaction network (**Figure 5C**) consisted of 38 nodes, representing 36 differential metabolites and interactions with two AMF genera. It comprised 94 pairs of interactions, with 59 pairs showing positive correlations and 35 pairs displaying negative correlations. The root exudate with the highest number of interactions in this network was N-decanoyl-L-Homoserine lactone (sugar). Finally, the maize intercropping interaction network (**Figure 5D**) consisted of 40 nodes, 37 differential metabolites, and interactions involving three AMF genera. It encompassed 120 pairs of interactions, with 99 pairs demonstrating positive correlations and 21 pairs exhibiting negative correlations. The root exudates with the most interactions in this network were N-methyl-Gabapentin (organic acid), 3,7,8. 15-Scirpenetetrol (sugar), and D-Pantothenic acid (organic acid). These findings indicated that intercropping significantly enhanced the mutualistic relationship between root exudates and the AMF community.

**Figure 6:**
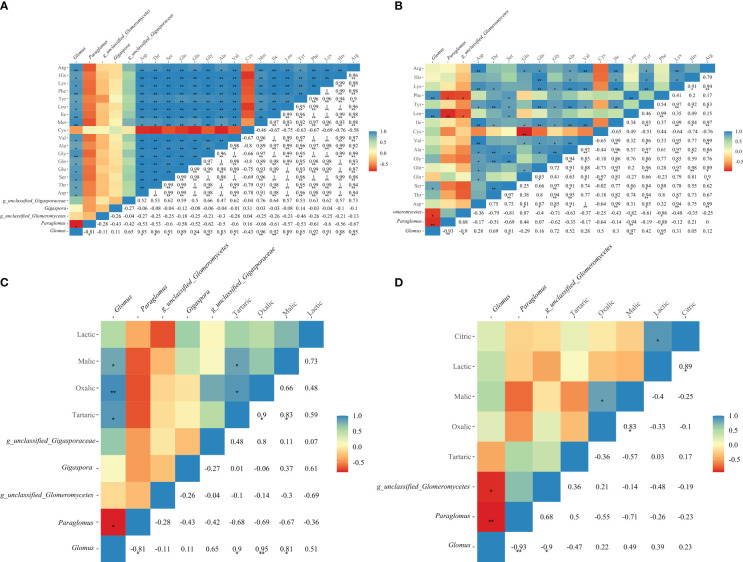
Correlation analysis between amino acids, organic acids and AMF community in soybean **(A, C)** and maize **(B, D)** soil.

Phenylalanine and leucine in maize root exudates exhibited a highly significant positive correlation with *Glomus*, the prominent genus in AMF (*P* < 0.01). Additionally, serine was positively correlated with *Glomus* (*P* < 0.05), as depicted in **Figure 6B**. In the case of soybean root exudates, glycine, methionine, isoleucine, phenylalanine, and arginine demonstrated significant positive correlations with *Glomus* (*P* < 0.01), as shown in **Figure 6A**. Furthermore, aspartic acid, threonine, serine, glutamic acid, and other metabolites displayed significant positive correlations with *Glomus* as well (*P* < 0.05). Interestingly, significant or highly significant correlations were observed between tartaric acid, oxalic acid, malic acid, and Glomus in soybean root exudates (*P* < 0.05), as displayed in **Figures 6C, D**.

## 4 Discussion

### 4.1 Different expression of root exudates under different planting modes

Root systems release a diverse array of organic substances into the rhizosphere, playing crucial roles in nutrient and water uptake, and mediating interactions with surrounding plants (Bais et al., 2006). These exudates range from low-molecular-weight entities like sugars and amino acids to high-molecular-weight compounds including proteins and polysaccharides (Bertin et al., 2003; Cesco et al., 2010). Notably, the nature of these exudates is often altered by different agricultural practices such as intercropping. For example, pairing maize with peanuts significantly modifies the latter’s root exudates, notably increasing total isoflavones by 22.4% (Gao et al., 2022). In a tea-persimmon intercropping system, intercropping was found to enhance the ability of roots to secrete amino acids (Zhu et al., 2006). Similarly, in a perilla/corn intercropping pot experiment, Qin et al. discovered that intercropping increased the concentration of oxalic acid and citric acid in the root exudates of perilla by 43.8% and 75.4%, respectively, while maize decreased them by 18.5% and 18.7%, respectively. Furthermore, the changes in root exudates were found to impact the bioavailability of soil Cd (Qin et al., 2021). Our findings echo these observations, demonstrating significant alterations in the composition and content of root exudates under intercropping conditions for both maize and soybean. Specifically, intercropped soybean and maize exhibited variations in 41 and 39 metabolites, respectively, compared to their monocropped counterparts, encompassing esters, sugars, amino acids, and organic acids. Notably, amino acid and organic acid levels in intercropped soybean were observed to be 3.61 and 1.80 times higher, respectively, than in their monocropped versions. The greater effect of intercropping on soybean may be due to the distinctive nutrient absorption dynamics between soybean and maize roots within intercropping systems (Li et al., 2007). For example, when intercropped leguminous crops with Poaceae crops, leguminous crops can produce more organic acids compared to monoculture, thereby activating insoluble phosphorus compounds. However, Poaceae did not show this reaction. This mainly because the competition of Gramineae roots for phosphorus (P) in rhizosphere soil of leguminous crops is fierce, which makes the concentration of available phosphorus in rhizosphere of leguminous crops decrease. As a result, leguminous crops produce more organic acids, activate soil insoluble phosphorus compounds, and promote the absorption of phosphorus (P) by leguminous crops (Li et al., 2007).

This study revealed that intercropping soybean and maize notably enhanced the rate of amino acid secretion by soybean roots (**Table 1**). This increase in secretion could be attributed to the promotion of nitrogen absorption and utilization capacity of soybeans as a result of intercropping (Duchene et al., 2017; Lu et al., 2024). Consequently, the root’s ability to secrete amino acids is enhanced. Amino acids present in root exudates serve as a vital nitrogen source for the growth of rhizosphere microorganisms (Broeckling et al., 2008). Intercropping, by enhancing amino acid secretion, provides a material foundation for increasing the abundance of rhizosphere microorganisms. Furthermore, the reciprocal influence between these microorganisms and the secretion of plant amino acids necessitates further investigation (Phillips et al., 2004).

Intercropping soybean and maize can lead to nutrient competition, potentially increasing the rate of organic acid secretion. Such secretion patterns often alter in response to nutrient deficiencies (Degryse et al., 2008). In this study, the primary influencing factors are light and nutrient stress caused by maize’s shading and competition for nutrients with soybean in the intercropping system (Li et al., 2009). Consequently, the types and secretion rates of organic acids may differ between monocropping and intercropping systems. Maize-soybean intercropping not only impacts the types of root organic acid secretion but also significantly enhances the rate of secretion. This can be attributed to the promotion of crop nitrogen (N) and phosphorus (P) absorption and utilization, improvement in crop carbon and nitrogen assimilation capacities, enhancement of organic acid metabolism in plants, and ultimately, alterations in the secretion characteristics of low-molecular-weight organic acids in the roots of intercropped crops.

### 4.2 Effects of root exudates on plant growth in intercropping system

Root exudates serve as vital agents for material and information exchange between plants and the soil, acting as the primary regulators of root activity (Gao et al., 2022). In this experiment, the soybean and maize under nylon netting separation, exhibited increased above-ground and below-ground biomass (**Figure 4B**; **Supplementary Figure S4**) as well as root growth (**Figures 4D–G**) compared to plastic sheeting separation. When separated by plastic, crops underground growth mode is equivalent to monoculture, and there is no competition in water and nutrient absorption caused by mass flow diffusion between crop roots, and there is also a lack of mechanisms and motivation to promote root growth (Peng et al., 2017). While under nylon mesh separation, Crops can interact through root exudates between each other. This implies that root exudates have a promoting effect on plant and root growth under the same aboveground condition. Furthermore, the direct addition of exogenous root secretions significantly boosted biomass and root growth in both soybean and maize (**Supplementary Figures S3A, B, F–M**), which was consistent with the results of the current study (Li et al., 2016). And a study has shown that L-Tryptophan in root exudates can promote the growth of roots (Lu et al., 2024). This finding suggested the essential role of root exudates on plants growth in soybean/maize intercropping systems. Additionally, the addition of root exudates led to an elevation in the number of soybean nodules during crop growth (**Supplementary Figure S3E**). This aligns with findings from Li et al (Li et al., 2008), which highlighted the improved nodulation and nitrogen-fixing capabilities in legumes when intercropped with cereals. Additionally, the legume/cereal intercropping model has been noted for its ability to enhance phosphorus (P) utilization by inducing the secretion of organic acids from leguminous roots (Latati et al., 2014), and for boosting nitrogen uptake through increased nodulation in leguminous plants (Fang et al., 2006; Zhao et al., 2020). Thus, root exudates in intercropping systems enhance the absorption and utilization of mineral elements by plants and enable their adaptation to the changes in the external environment.

This study found that intercropping significantly increased the secretion of aspartic acid, glycine, alanine, tyrosine and glutamic acid (**Table 1**), which may promote the growth of crops. It has been found that soil amino acids were closely related to soil fertility and plant productivity (Moreira and Moraes, 2016), and affect the metabolism of carbon and nitrogen in the soil. A study found that glutamic acid plays a crucial role as an important component in chlorophyll synthesis, significantly promoting crop growth (El-Metwally et al., 2022). When glutamic acid is present in the free form in soil pore water, it forms a film on the surface of crop root hair (Limami et al., 2008), effectively enhancing nutrient absorption from the soil and regulating the acidity and alkalinity of the soil microbial environment (Limami et al., 2008). In this research, we found that root exudates from soybean and maize, when intercropped, play pivotal roles in essential metabolic pathways associated with amino acid metabolism, significantly influencing carbon and nitrogen dynamics within the soil (**Figure 3**). Among these, α-linolenic acid metabolism was critical for enhancing crop quality. Metabolism of compounds such as alanine, aspartate, and glutamate is crucial in modulating nitrogen flow, augmenting carbon availability, and altering cytosolic pH levels to support plant development (Limami et al., 2008).In maize, nitrogen metabolism stands as a critical pathway reflecting not only the plant’s developmental stages but also affecting yield and quality (Zhao et al., 2018; Lu et al., 2019). Compounds present in root exudates are involved in significant pathways of amino acid and carbon and nitrogen metabolism, potentially explaining the improved mycorrhizal rate, enhanced nutrient absorption efficiency, and increased crop biomass after intercropping (Moe, 2013). Additionally, in soybean and maize intercropping systems, elements such as sugars, carboxylic acids, and amino acids act as primary carbon sources for rhizospheric microorganisms (Dong et al., 2013). Intercropping systems also increased the secretion of organic acids from plant roots, especially legumes (**Table 2**). Organic acids are instrumental in nutrient enrichment within the root vicinity, easing nutrient acquisition by plants and thereby enhancing their nutritional profile (Haichar et al., 2014). These acids are crucial for activating essential nutrient elements, with oxalic, citric, acetic, succinic, tartaric, malic, and malonic acids being notably prevalent in root exudates. Their presence reduces rhizospheric pH, boosting the solubility of compounds otherwise insoluble (Shen et al., 2002), and forms chelates with various metal ions (Dakora and Phillips, 2002), thus liberating nutrient in the rhizosphere soil. The augmentation in organic acid exudation within the intercropping arrangement is thought to play a crucial role in mobilizing non-soluble soil nutrients, particularly enhancing phosphorus (P) solubility (Jones, 1998), thereby boosting the accessible phosphorus (P) levels in the rhizospheric soil and fostering an improved nutritional state through natural self-adjustment mechanisms. Furthermore, these organic acids can react with toxic metal ions in the soil, forming compounds with reduced toxicity and mitigating the harmful effects of toxic ions on plant roots. Notably, this study identified two main types of root exudates, namely amino acids and organic acids, each exerting positive effects on crop growth and soil environment, aligning with the experimental results obtained.

### 4.3 Effects of root exudates on soil environment and microorganisms, especially AMF, under intercropping system

In the intercropping rhizosphere environment, plants primarily utilize root exudates to regulate the physical and chemical properties of the soil (Bertin et al., 2003; Okubo et al., 2016). Our study demonstrates a significant correlation between the type and content of root exudates and the levels of AN, AP, and AK in intercropped soils (**Supplementary Figure S6**). Root exudates in intercropping systems play a crucial role in activating otherwise inaccessible nutrients in the soil, enhancing nutrient effectiveness, and optimizing root distribution for resource utilization (Li et al., 2006, 2014). Through the secretion of root exudates, plants can also improve soil microbiological conditions, alleviate nutrient stress, and regulate microbial communities to promote their growth (Withers et al., 2020). Moreover, root exudates serve as substrates, signaling molecules, and antimicrobial agents, which influence the relative abundance of rhizosphere microorganisms and indirectly enhance the soil environment by stimulating microbial growth and activity, facilitating the production of extracellular enzymes (Li et al., 1997; Nardi et al., 2002), and promoting the degradation of soil organic matter (Razavi et al., 2016; Lopez-Sangil et al., 2017).

Root exudates not only regulate the nutrient availability of the rhizosphere soil but also influence and modulate the soil microbial community (Philippot et al., 2013; Zhalnina et al., 2018). Intercropping significantly affects the structure and diversity of the soil microbial community through root exudates (Chen et al., 2019). Our study found that intercropping increased AMF colonization and improved AMF community structure (**Figure 4C**; **Supplementary Figure S5**). These findings align with a previous field experiment that demonstrated how intercropping substantially improves the diversity of mycorrhizal fungi (Zhang et al., 2020). Moreover, our study revealed significant differences in root exudates between soybean/maize monocropping and intercropping, and correlation analysis indicated a close relationship between root exudates and AMF (**Figure 5**). Furthermore, the addition of soybean or maize root exudates to crop growth substantially enhances mycorrhizal fungi colonization (**Supplementary Figures S3C, D**). Root exudates are crucial in modulating plant-AMF interactions and shaping the soil microbial community (Haichar et al., 2008; Hugoni et al., 2018). Studies demonstrate that the addition of root exudates significantly stimulates the germination rates of AM fungal spores and promotes hyphal growth (Bücking et al., 2008; Nagata et al., 2016). This phenomenon can be attributed to the metabolic function of root exudates and the reciprocal regulation between exudates and soil microorganisms. Research shows that roots release various compounds during plant growth, such as sugars, organic acids, amino acids, phenolic acids, and other metabolites. These compounds selectively attract bacteria and fungi, promoting their aggregation and proliferation in the rhizosphere, thus forming a rhizosphere interaction (Brencic and Winans, 2005). Root exudates are key in initiating and modulating interactions between plant roots and soil microbes, crucially influencing the communication between these two entities (Bais et al., 2006; Houlden et al., 2008). Even minor variations in the composition or quantity of these exudates can significantly alter the microbial community and its structure in the rhizosphere (Ling et al., 2011). Accumulating evidence have shown that a range of compounds including vitamins, amides, terpenoids, organic acids, amino acids, derivatives, lipids, and benzoxazinoids play roles in regulating both the soil microbiota and plant growth (Faure et al., 2009; Hu et al., 2018; Zhalnina et al., 2018). Moreover, components like sugars, organic acids, amino acids and derivatives, and lipids serve as carbon and energy sources for rhizosphere microorganisms (Mavrodi et al., 2021). Our study revealed that intercropping between soybean and maize increased the secretion of sugar, amino acids, organic acids, and lipids in root exudates. This enhanced secretion provides an abundant supply of carbon sources for rhizosphere microorganisms, thereby promoting the improvement of rhizosphere microbial diversity, particularly AMF diversity in intercropping systems. Additionally, we identified important metabolic pathways in soybean/maize intercropping, which involved amino acid metabolism, carbon and nitrogen metabolism, carbon fixation by photosynthetic organisms, and other important pathways (**Figure 3**). The increase in soil carbon and nitrogen metabolism may be a significant factor contributing to the enhanced mycorrhizal colonization rate following intercropping. Furthermore, the carbon fixation pathway of photosynthetic organisms exhibits a functional connection with AMF (Jeewani et al., 2021; Agnihotri et al., 2022). Organic acids present in root exudates, as vital energy sources for soil microbial growth, stimulate microbial and enzyme activities in the root zone (Haichar et al., 2014). One previous study has demonstrated that organic acids, such as fumaric acid, promote the formation of biofilms in Bacteroides polymyxa SQR9 (Liu et al., 2014). In our investigation, we detected oxalic acid, malic acid, citric acid, succinic acid, and tartaric acid in the root exudates of maize/soybean intercropping, which may serve as nutritional sources for the growth and reproduction of AMF. Furthermore, organic acids can activate insoluble nutrients in the soil and improve its physical and chemical properties, thereby enhancing mycorrhizal colonization and AMF diversity. We also observed a close relationship between the relative abundance of the significant AMF genus *Glomus_f_Glomerace* and the concentration of differential metabolites (**Figures 5**, **6**). Studies show that *Glomus*, can effectively obtain nitrogen (N) and phosphorus (P) (Jiang et al., 2020), and *Glomus* is beneficial to carbon fixation and soil organic carbon protection, therefore *Glomus* plays an important role in soil nutrient cycle. Intercropping system can increase the abundance of *Glomus* in AMF community, which is of positive significance to improve soil fertility. Correlation analysis revealed significant associations between several differential metabolites (tartaric acid, oxalic acid, malic acid, aspartic acid, and alanine) and the AMF community (**Figure 6**). Notably, the photosynthetic carbon fixation pathway involving aspartic acid was identified as a pathway that may be related to the formation of AMF (Agnihotri et al., 2021), and aspartic acid showed a significant connection with *Glomus_f_Glomerace* (*P* < 0.05, **Figure 6**). Therefore, it is demonstrated that the root exudates of soybean/maize intercropping mediate changes in AMF diversity and community structure. Moreover, the mycorrhizal symbiotic relationships between AMF and plants can enhance the nutrient absorption capacity and help to improve the growth and productivity of host plants (Ferrol et al., 2019). Therefore, root exudates in intercropping can not only directly promote crops growth, but also indirectly promote plant growth by regulating the colonization and community of AMF. Intercropping can bring higher crop productivity as well as maintain soil nutrients, which is of positive significance to improve the sustainability of soil and the diversity and stability of farmland ecosystem. Therefore, it is summarized that in the intercropping system of soybean/maize, intercropping influences AMF and soil microecology by altering root exudate patterns, ultimately establishing intercropping dominance.

## 5 Conclusion

In this study, the diversity of AMF communities in the maize/soybean intercropping system was distinctly influenced due to altered root exudate compositions and metabolic profiles. Notably, analyses revealed 39 unique metabolites in the exudates of intercropped versus monocropped maize, and 41 distinct metabolites between intercropped and monocropped soybean (*P* < 0.05, VIP > 1). There was a notable increase in the release of amino acids, organic acids, and other substances in intercropped system (*P* < 0.05). Such changes were associated with enhanced growth in plants and roots, increased mycorrhizal colonization, and improved soil nutrient status, which was attributed to the influence of root exudates. And the addition of root exudates significantly bolstered plant growth and AMF colonization in both soybeans and maize. Crucial metabolic pathways involved in differential metabolites, such as amino acid metabolism and carbon fixation pathways, were found to be significantly correlated to AMF functionality. The variation in root exudates and metabolic processes contributed to improved plant growth and soil microecology. Metabolites like tartaric acid, oxalic acid, malic acid, aspartic acid, and alanine showed significant correlations with AMF communities (*P* < 0.05). Notably, the pathway of photosynthetic carbon fixation involving aspartic acid demonstrated a close functional relationship with AMF, and aspartic acid was significantly associated with the dominant AMF genus, Glomus_f_Glomerace. The findings suggested that maize/soybean intercropping was more effective in increasing AMF colonization and metabolite accumulation than monocropping. Compounds such as aspartic acid in the root exudates of the intercropping system significantly altered the diversity and community structure of AMF, thus contributing to the improvement of soil quality and microecology. This research sheds light on the pivotal role of root exudates in facilitating plant-soil interactions within intercropping systems and underscores their importance in sustaining agricultural ecosystem health and promoting soil vitality. Additionally, it provides a scientific basis for diverse cropping aimed at conserving black soil and ensuring sustainable field management.

## Data availability statement

The data presented in the study are deposited in the NCBI Sequence Read Archive repository, accession number Bio Project PRJNA781197.

## Author contributions

SZ: Data curation, Formal analysis, Investigation, Validation, Writing – original draft. SL: Funding acquisition, Writing – review & editing, Project administration, Supervision. LM: Funding acquisition, Writing – review & editing. XL: Writing – review & editing. YZ: Visualization, Writing – review & editing. SCZ: Writing – review & editing, Software. HZ: Writing – review & editing.
