# Supplementary material for: Root exudation under maize/soybean intercropping system mediates the arbuscular mycorrhizal fungi diversity and improves the plant growth
Source: Front Plant Sci. 2024 Jun 14;15:1375194. doi: 10.3389/fpls.2024.1375194 (PMC11211593; doi:10.3389/fpls.2024.1375194)
Supplement: Supplementary file 1 [file DataSheet_1.doc]

***Supplementary materials***

This supporting information contains 6 pages document including 6 figures.


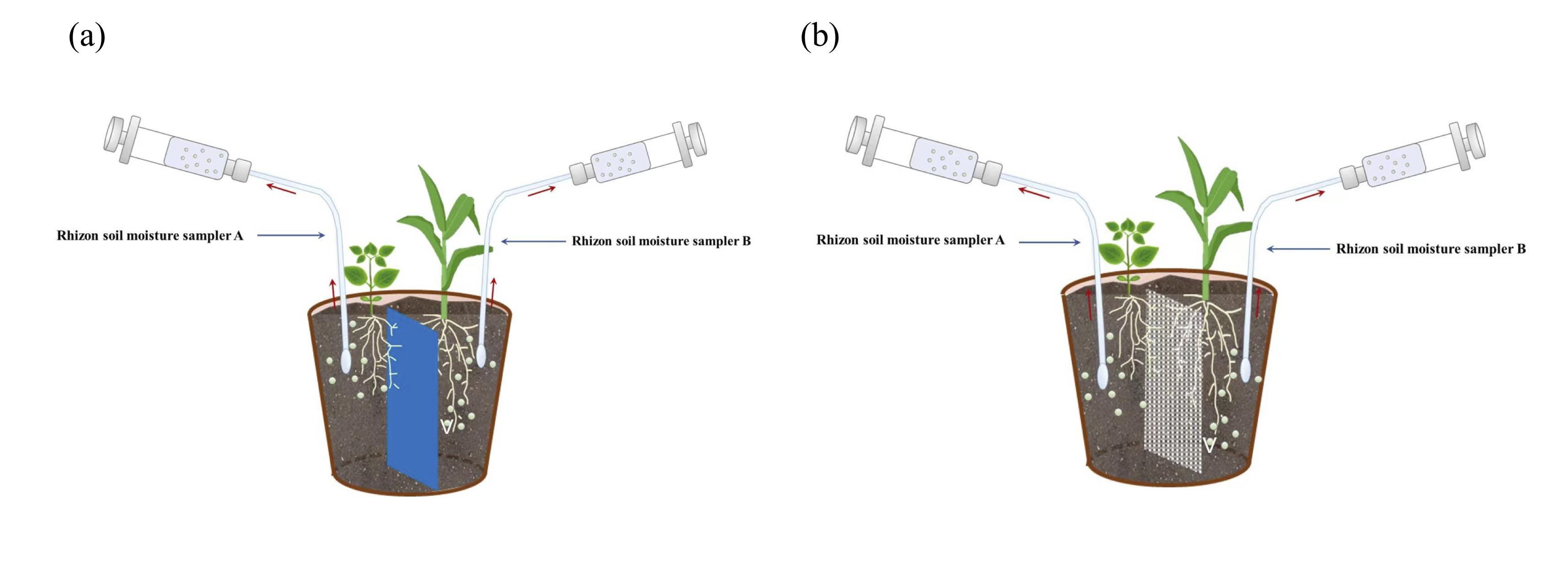


**Figure S1** Schematic diagram of separating the root systems of soybean and maize by the solid root barrier(a), nylon mesh(b) in experiment 1.

**
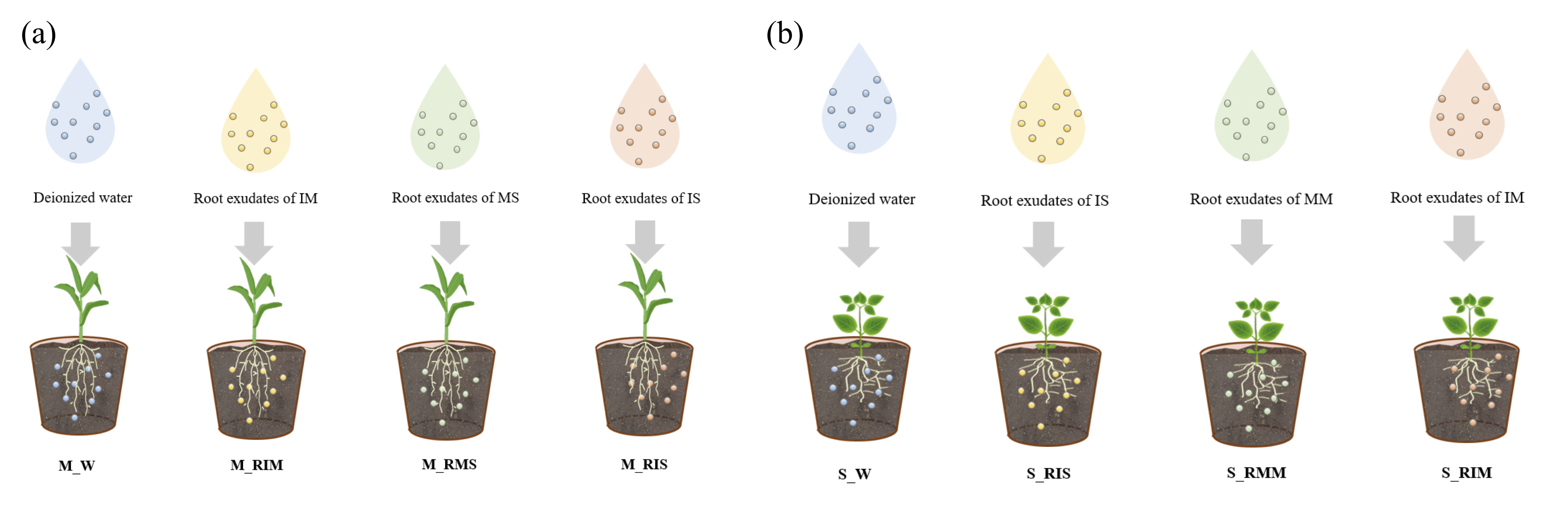
**

**Figure S2** Schematic diagram of experiment 2.


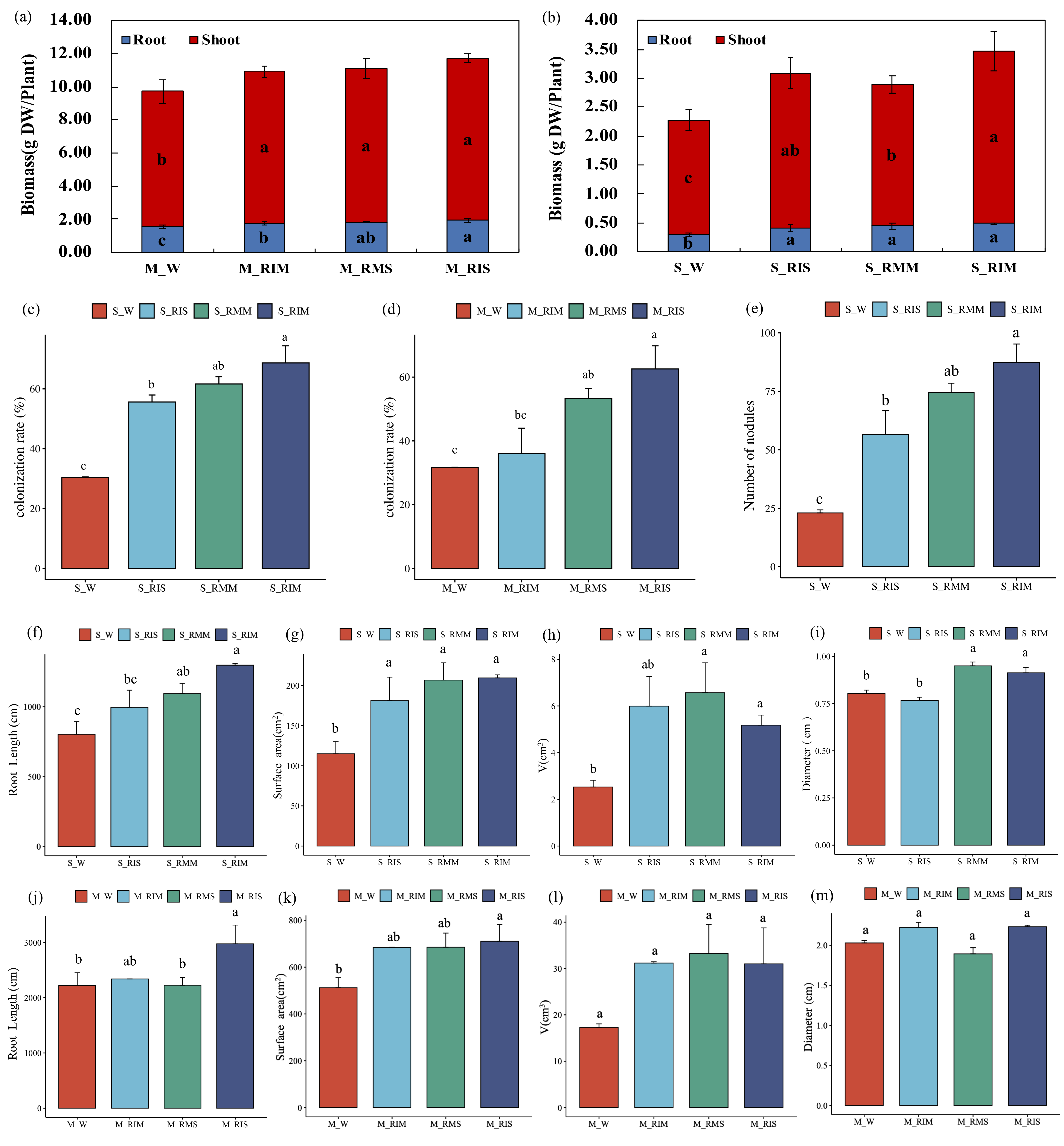


**Figure S3** Effects of added root exudates on aboveground and belowground biomass of maize **(a)** and soybean **(b)**. Effects of added root exudates on myorrhizal colonization rate of soybean **(c)** and maize **(d)**. Effects of added root exudates on the number of root nodules in soybean **(e)**. Effects of added root exudates on soybean root morphology **(f, g, h, i)** and maize root morphology **(j, k, l, m)**.

**Figure S4** Effects of different separating Patterns on plant growth (a), Effects of added root exudates on plant growth of maize (b).


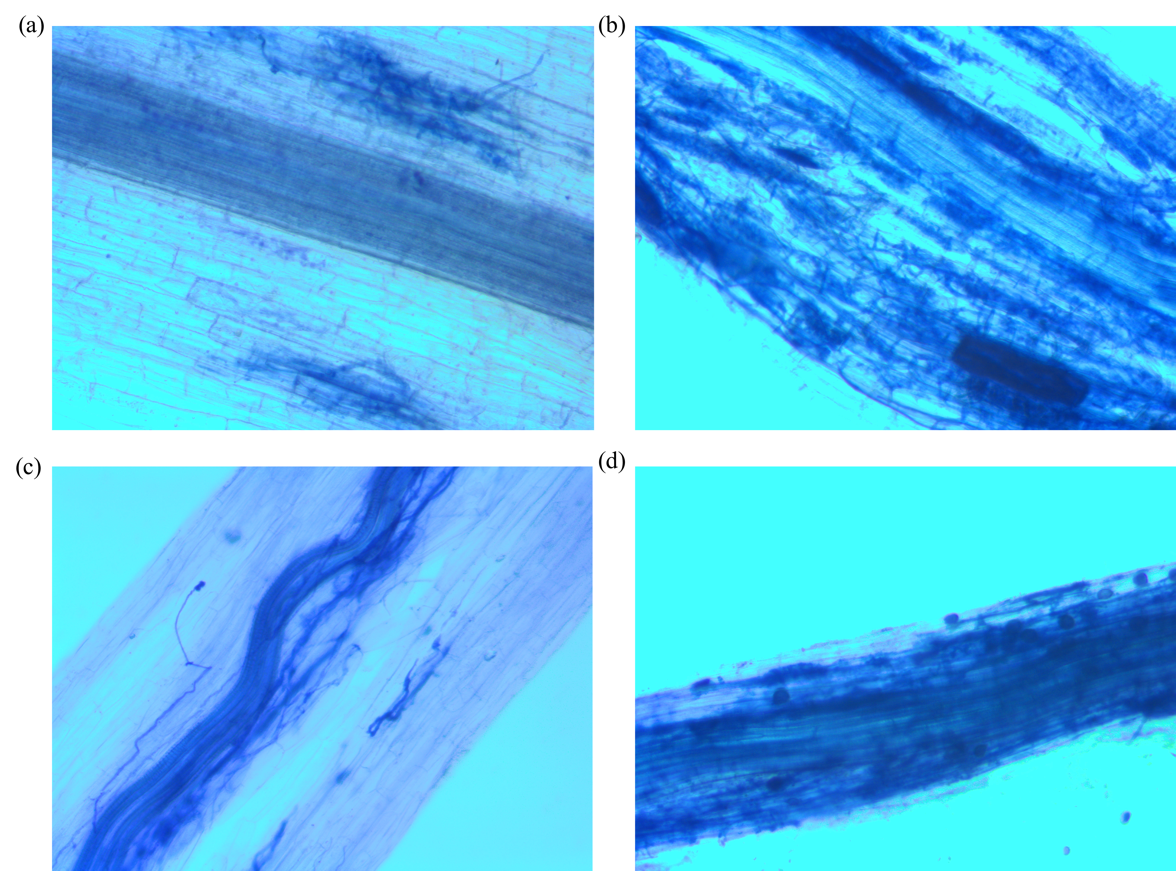


**Figure S5** Mycorrhizal infection of maize and soybean at different treatments. (a) monocropping maize (b) intercropping maize (c) monocropping soybean (4) intercropping soybean


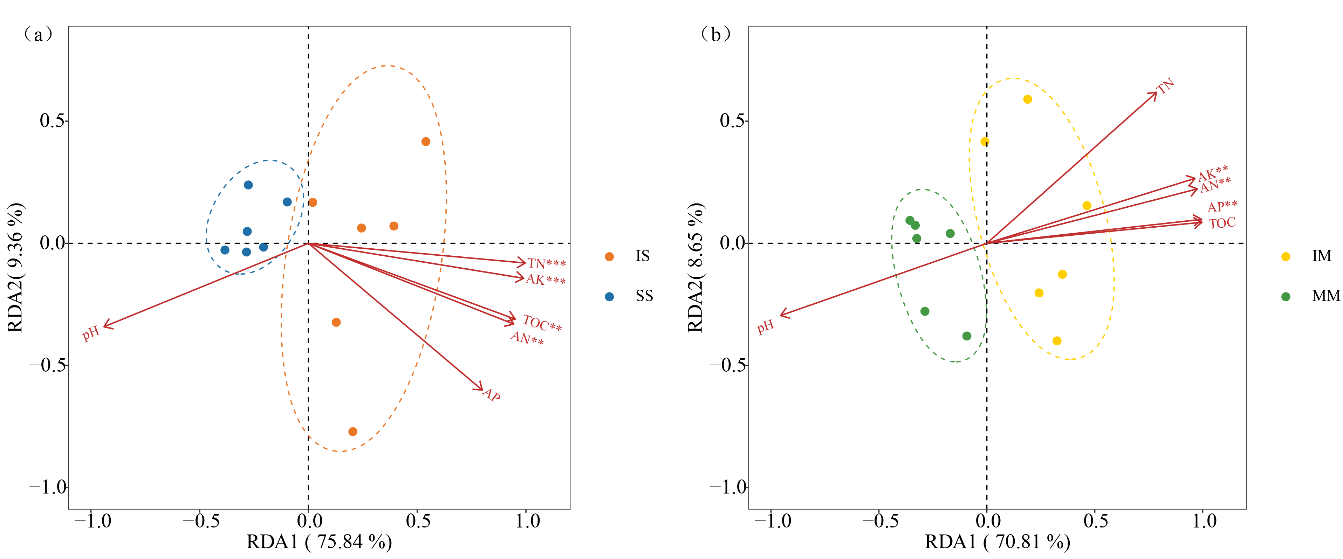


**Figure S6** the interactive response relationship between soil environmental factors (TN, TOC, AN, AP, AK, pH) and the composition of root exudates in various treatments（intercropping and monocropping）based on RDA analysis.
